# Early-onset restrictive eating disturbances in primary school boys and girls

**DOI:** 10.1007/s00787-014-0622-z

**Published:** 2014-10-09

**Authors:** Susanne Kurz, Zoé van Dyck, Daniela Dremmel, Simone Munsch, Anja Hilbert

**Affiliations:** 1 Department of Psychology, University of Fribourg, Rue P.-A. Faucigny 2, 1700 Fribourg, Switzerland; 2 Research Unit INSIDE, University of Luxembourg, Campus Walferdange, Route Diekirch, Walferdange, Luxemburg; 3 Integrated Research and Treatment Center Adiposity Diseases, University of Leipzig Medical Center, Philipp-Rosenthal-Strasse 27, Leipzig, Germany

**Keywords:** Avoidant restrictive food intake disorder, Early-onset restrictive eating disturbances, Middle childhood, Prevalence

## Abstract

This study sought to determine the distribution of early-onset restrictive eating disturbances characteristic of the new DSM-5 diagnosis, avoidant/restrictive food intake disorder (ARFID) in middle childhood, as well as to evaluate the screening instrument, Eating Disturbances in Youth-Questionnaire (EDY-Q). A total of 1,444 8- to 13-year-old children were screened in regular schools (3rd to 6th grade) in Switzerland using the self-report measure EDY-Q, consisting of 12 items based on the DSM-5 criteria for ARFID. 46 children (3.2 %) reported features of ARFID in the self-rating. Group differences were found for body mass index, with underweight children reporting features of ARFID more often than normal and overweight children. The EDY-Q revealed good psychometric properties, including adequate discriminant and convergent validity. Early-onset restrictive eating disturbances are commonly reported in middle childhood. Because of possible negative short- and long-term impact, early detection is essential. Further studies with structured interviews and parent reports are needed to confirm this study’s findings.

## Introduction

Eating disturbances in middle childhood are common; however, their assessment and presentation remain under debate. According to the former Diagnostic and Statistical Manual of Mental Disorders Fourth Edition (DSM-IV-TR), about 40–60 % of children with an eating disorder are diagnosed with an eating disorder not otherwise specified [1–6]. Recently, the fifth revision of the Diagnostic and Statistical Manual (DSM) has included the category avoidant/restrictive food intake disorder (ARFID) [7] to capture the key clinical features of avoiding or restricting food intake without distorted cognitions about weight and shape. ARFID is defined as a feeding or eating disturbance associated with significant clinical and/or psychosocial impairment, which can be present across the lifespan [7]. It also serves as a replacement for the DSM-IV-TR diagnosis of feeding disorder of infancy and early childhood. The Eating Disorders Consultation Group for revision of the International Classification of Diseases (ICD-10; [8] ) also proposed ARFID as one of 6 main categories of “feeding and eating disorders” [9]. ARFID includes several presentations described in middle childhood which do not fit any previous eating disorder diagnosis [10]. They can be characterized in terms of a lack of interest in eating or food, eating a very limited amount of food connected to sensory or other properties of the food (colour, texture, taste, brand), and the avoidance of food based on specific fears, such as choking or vomiting. There is an abundance of terms describing these presentations (e.g. food avoidance emotional disorder, selective eating, picky eating, restrictive eating, sensory food aversion, food neophobia, functional dysphagia). With the inclusion of ARFID as a diagnosis on its own, the clinical utility and understanding of such presentations should be improved.

Among the few epidemiological studies on early-onset restrictive eating disturbances, Cooper and colleagues [11] conducted a systematic assessment in 8- to 18-year-old eating disordered patients referred to specialist child and adolescent eating disorder services, using diagnostic interviews. Of the 86 patients, 29 % were identified with food avoidance emotional disorder, 17 % with selective eating, and 1 % with functional dysphagia, applying the Great Ormond Street criteria (GOS [12] ). These presentations would all fall into the diagnosis of ARFID. A recent study by Ornstein and colleagues [13] compared eating disorders in 215 8- to 21-year-old patients using the DSM-IV and the proposed DSM-5 criteria and found 14 % of the cases to fulfil the criteria for ARFID. Similarly, in a retrospective case–control study with 712 8- to 18-year-olds with restrictive eating [14], 13.8 % were identified with ARFID using DSM-5. In a national surveillance study in Canada [4], the incidence and age-specific clinical presentation of early-onset eating disorders were assessed using paediatric reports on 5- to 12-year-old children, showing an incidence of 2.6 cases per 100,000 person-years. Only 62.1 % of the cases met criteria for anorexia nervosa based on DSM-IV-TR and the authors concluded that the other cases would likely fulfil the criteria for ARFID. In a British national surveillance study ( <13 years of age), the overall incidence of early-onset eating disorders with modified DSM-IV criteria was 3.01 cases per 100,000, using questionnaires completed by paediatricians or child psychiatrists [2]. Of those, 37 % met criteria for anorexia nervosa, 1.4 % for bulimia nervosa, 43 % for eating disorder not otherwise specified, and 19 % presented with food avoidance and underweight without shape or weight concern.

Thus, epidemiological studies on ARFID present hitherto heterogeneous results in various clinical samples, ranging from 13–29 % of children and adolescents presenting to child and adolescent eating disorder services. To our knowledge, no study on the prevalence of ARFID features in the general non-clinical population has been conducted. As early-onset restrictive eating disturbances are often accompanied by a higher risk of developing eating disorders in the future [15–18] and tend to show worse long-term outcome [19, 20], early detection is essential for targeted prevention and early intervention. To identify eating disturbances early on, reliable screening instruments are essential [21]. Therefore, the Eating Disturbances in Youth-Questionnaire (EDY-Q; [22] ) with 12 items on ARFID was developed. In this context, the goal of this study was to determine the distribution of ARFID symptoms in middle childhood by self-report, as well as to validate the EDY-Q.

## Method

### Recruitment and sample

Children between 8 and 13 years of age were recruited from regular schools (3rd–6th grade) in Switzerland in the cities of Fribourg, Lausanne, Bern, and their surrounding areas. The screening was part of the Swiss University Study of Nutrition (SUN; Hilbert & Munsch, 2010), which was approved by the Ethics Committees of the Canton of Fribourg, as well as of the Department of Psychology of the University of Fribourg.

The languages of the children participating in this study were German or French. Schools of all socioeconomic backgrounds were included. Informed consent was sought by the cantonal board of education, the school board and by the parents, who gave their children permission to fill out the questionnaires. Several self-report questionnaires were used, including the Eating Disorder Examination-Questionnaire adapted for Children (ChEDE-Q [21, 23], German version [24], French version Dremmel et al., in preparation), Conners ADHD–Index–Self-Report Form (Conners 3AI^TM^ [25], German Version Lidzba, in preparation, French version Dremmel et al., in preparation) and the newly developed EDY-Q (EDY-Q; German and French versions [22]). The French version was established using forward- and back-translations. Children were told to ask the researcher for help if they had problems understanding the questions.

A total of 1,452 children were screened. 5 of them did not complete the EDY-Q and another 3 filled out less than 40 % of the questionnaire, thus were excluded from analysis. This resulted in a total of 1,444 children, of which 681 (47.2 %) completed the German and 763 (52.8 %) the French version of the EDY-Q. The sex distribution was relatively even with 665 (46.1 %) boys and 779 (53.9 %) girls, and the mean age was 10.55 years (SD = 1.89). The body mass index (BMI; kg/m^2^) was calculated from the children’s subjective estimation of height and weight, according to German reference scores [26] and resulted in a mean BMI of 17.23 (SD = 2.60) and a mean BMI standard deviation (BMI SDS) score of −0.23 (SD = 1.20). According to the guidelines of the Workgroup for Adiposity in Childhood and Adolescence [27], 13 % of participants were underweight (187/1,356; BMI < 10th BMI percentile), 75.3 % were normal weight (1,087/1,356; 10th–90th BMI percentile), 4 % were overweight (58/1,356 > 90th BMI percentile), and 1.7 % were obese (24/1,356 > 97th BMI percentile). Because of missing data on weight or height, the BMI could not be calculated for 6.1 % of the children (81/1,444).

### Measures

To detect early-onset restrictive eating disturbances characteristic of ARFID, the self-report scale EDY-Q was developed [22]. The items were based on the diagnostic criteria for ARFID proposed by DSM-5 and on the GOS criteria, as well as on the literature on early-onset restrictive eating disturbances.

The EDY-Q consists of 14 items, including two questions on pica and rumination disorders, which were not reported in this study. The other 12 items cover three possible presentations of ARFID (food avoidance emotional disorder, selective eating, and functional dysphagia) and include two items on weight and shape concern as important exclusion criteria of ARFID [7]. For each item, a 7-point Likert scale was used (never = 0; always = 6). First results on a smaller subset of this study’s sample (*N* = 730) revealed a four-factor solution with clear allocation of all items to the scales and good item characteristics but low internal consistencies [22].

Items of the EDY-Q that best represented the DSM-5 diagnostic criteria for ARFID were extracted for prevalence estimation. The EDY-Q diagnostic items for ARFID include three items on the appearance of the food avoidance or restriction (Interest in Food, Sensory Food Avoidance, Fear of Choking), one item on the failure to meet adequate weight growth (Underweight), and two items on the exclusion criteria for ARFID (Weight and Shape Concern). Further, cut-off criteria were defined for the EDY-Q diagnostic items for ARFID. To fulfil the criteria for ARFID, the child had to report the equivalent eating behaviour at least “often” (cut-off ≥ 4), which is a rather high cut-off score, indicated for self-report questionnaires measuring eating disturbances [28]. Items were dichotomized and were recoded into “no”, for scores between “never” (score = 0) and “sometimes” (score = 3) or into “yes”, for scores between “often” (score = 4) and “always” (score = 6). Additionally, distorted cognitions on weight and shape as exclusion criteria had to be reported less than “sometimes” (cut-off < 3) to fulfil the criteria for ARFID. Furthermore, an EDY-Q mean score was calculated with all items except for the two items on the exclusion criteria. These were calculated separately, which allowed us to calculate convergent and divergent validity of the EDY-Q. The EDY-Q mean score was calculated to get additional information on ARFID related behaviour as well as for psychometric analyses.

Measures of BMI, as well as several items of the ChEDE-Q, were used for validation of the EDY-Q. Overall, the ChEDE-Q has good internal consistencies (α = 0.94) and good discriminant and convergent validity [24]. The following associations were hypothesized: no correlations between the ChEDE-Q items (Restraint over Eating, Fear of Weight Gain, and Dissatisfaction with Weight) with the EDY-Q diagnostic items for ARFID and the EDY-Q mean score, as these ChEDE-Q items cover exclusion criteria for ARFID; positive correlations between the ChEDE-Q items with the EDY-Q items of the exclusion criteria for ARFID. Regarding group differences, the EDY-Q was assumed to differentiate between underweight versus normal- and overweight children.

### Statistical analysis

Psychometric analysis addressed missing values, item difficulty index (e.g. [29]), item-total correlation, as well as the Kolmogorov–Smirnov test for testing normal distribution of items. Cronbach’s alpha was used to determine internal consistency of the EDY-Q mean score.

To determine discriminant, divergent, and convergent validity, items of the EDY-Q were correlated with items of the ChEDE-Q using Spearman’s Rank correlation coefficient. Furthermore, BMI groups were compared using Kruskal–Wallis rank sum tests and post hoc analyses using Bonferroni corrections. After prevalence estimation of ARFID using the dichotomized EDY-Q diagnostic items for ARFID, the distribution of ARFID, as well as the EDY-Q mean score difference in sex, age, or BMI category was tested with Chi square and Kruskal–Wallis rank sum tests as well as post hoc analyses using Bonferroni corrections.

A two-tailed alpha level of ≤0.05 was used for all statistical analyses. Analyses were conducted using SPSS 20.0.0 (SPSS Inc., Chicago, IL, USA).

## Results

### Psychometric analyses

Missing data were low with 0–1.0 % for each item in the total sample. Over half of the items (55.6 %) were answered with 0 = “never”. The Kolmogorov–Smirnov test was significant for all items (*p* < 0.001) with a significantly left skewed and high kurtosis distribution (Table 1). Item difficulty was medium to high [29], ranging from 0.05 ≤ *p*_m_ ≤ 0.38 (Table 1). Thus, items had a low probability of high scores. Corrected item-total correlations were low to medium (0.23 ≤ *r*_it_ ≤ 0.39) (Table 1). Internal consistency for the EDY-Q mean score was acceptable (Cronbach’s *α* = 0.62).

**Table 1:** Item distribution *(N* = 1,444*)*

EDY-Q items	*M*	SD	Skewness	Kurtosis	*p*_m_	*r*_it_
			z-score	z-score		
Food avoidance						
Food avoidance	0.95	1.42	24.11	15.03	0.16	0.32
Interest in food	1.23	1.65	20.77	7.63	0.20	0.31
Emotional food avoidance	1.89	2.17	12.92	−5.80	0.32	0.28
Selective eating						
Selective eating behaviour	2.25	2.07	8.98	−7.20	0.38	0.25
Avoidance to try new foods	1.88	2.05	11.91	−5.68	0.31	0.33
Sensory food avoidance	1.89	1.98	12.47	−4.47	0.32	0.39
Functional Dysphagia						
Fear of choking	0.65	1.49	39.45	44.16	0.11	0.30
Fear of swallowing	0.28	0.98	66.68	153.02	0.05	0.25
Weight problems						
Underweight	1.22	1.87	22.45	6.70	0.20	0.30
Wish to gain weight	0.68	0.04	38.33	37.51	0.11	0.23

EDY-Q, Eating Disturbances in Youth-Questionnaire [22]; *M*, mean value; *SD,* standard deviation*; p*_m_, item difficulty indices; *r*_it_, item-total correlations

### Discriminant, divergent, and convergent validity

As hypothesized, the EDY-Q diagnostic items for ARFID were not correlated and the EDY-Q exclusion criteria were positively correlated with the ChEDE-Q items Restraint over Eating, Fear of Weight Gain, and Dissatisfaction with Weight. The EDY-Q mean score was positively, though weakly, correlated with the ChEDE-Q items (Table 2). Thus, divergent and convergent validity was demonstrated partially for items of the EDY-Q.

**Table 2:** Spearman Rank Correlations of EDY-Q diagnostic items for ARFID, EDY-Q mean score and EDY-Q exclusion criteria with ChEDE-Q Items (*N* = 1,444)

EDY-Q	Diagnostic items for ARFID	Exclusion criteria for ARFID	EDY-Q mean score
ChEDE-Q items	*r*_s_	*r*_s_	*r*_s_
Restraint over eating	−0.06*	0.41**	0.23**
Fear of Weight gain	−0.01	0.46**	0.21**
Dissatisfaction with weight	−0.05*	0.42**	0.22**
Self-induced vomiting	−0.04	0.09**	0.10**
Laxative misuse	−0.04	0.05	0.04

EDY-Q Eating Disturbances in Youth-Questionnaire [22];ChEDE-Q: Eating Disorder Examination-Questionnaire adapted for Children [23], *ARFID* Avoidant/Restrictive Food Intake Disorder, *r*_s_ Spearman Rank Correlation Coefficient

* *p* < 0.05, ** *p* < 0.01

Regarding discriminant validity, BMI groups differed significantly in the EDY-Q diagnostic items for ARFID (*H*(2) = 61.44, *p* < 0.001), as well as in the EDY-Q mean score (*H*(2) = 33.07, *p* < 0.001) (Table 3). According to post hoc comparisons, underweight children scored significantly higher on the EDY-Q diagnostic items for ARFID (*p* < 0.01) than normal- and overweight children. Further, underweight and overweight children scored significantly higher on the EDY-Q mean score (*p* < 0.01) than normal weight children. Therefore, discriminant validity was found for BMI status.

**Table 3:** Group differences for ARFID and the EDY-Q mean score *(N* = 1,444)

	EDY-Q diagnostic items for ARFID		EDY-Q mean score		
	%	χ^2^ (*df* = 1)	*M*	SD	*U* Test
Sex					
Boys	1.3	0.44	1.27	0.82	256,834.5
Girls	1.9		1.27	0.83	
Age					
7–10 years	1.5	0.56	1.35	0.86	225,054.5**
11–13 years	1.7		1.22	0.79	
BMI		(*df* = 2)	*M*	SD	*H Test*
Underweight	1.7	61.48**	1.55	0.89	33.07**
Normal weight	1.5		1.20	0.80	
Overweight	0.0		1.51	0.83	

*ARFID* Avoidant/Restrictive Food Intake Disorder, *EDY-Q* Eating Disturbances in Youth-Questionnaire, χ^2^ Chi square test, *U* Mann–Whitney test, *H* Kruskal–Wallis test, *M* mean value, *SD* standard deviation, *BMI* body mass index; underweight, <10th BMI percentile; normal weight, 10th–90th BMI percentile; overweight, >90th BMI percentile

** *p* < 0.01

### Prevalence

A total of 46 children (3.2 %) reported features of ARFID in the EDY-Q. Among these children, 18 (39.1 %) indicated a lack of interest in eating or food, 28 (60.9 %) children indicated a limited food intake due to the sensory properties of the food, and seven (15.2 %) children indicated a food avoidance based on negative consequences of eating, such as choking or vomiting. A total of seven (15.2 %) children reported at least two of these eating disturbances.

Underweight children reported features of ARFID significantly more often than normal weight children and the latter reported features of ARFID significantly more often than overweight children (Table 3). No significant distribution differences were found for sex and age regarding the self-reported features of ARFID. With respect to the EDY-Q mean score, under- and overweight children scored higher than normal weight children and younger children (7–10 years.) scored higher than older children (11–13 years.) (Table 3), but no distribution difference was found for sex.

Additional analyses of the differences between German versus French language showed similar results on the psychometric properties and distribution (details available upon request).

## Discussion

To our knowledge, this is the first investigation on the prevalence of early-onset restrictive eating disturbances characteristic of the new DSM-5 category ARFID in a non-clinical middle childhood population sample. Additionally, the screening questionnaire EDY-Q was validated, showing reasonably good validity.

ARFID reported features were relatively common (3.2 %) among 8–13-year-old children. Of the children reporting features of ARFID, restrictive eating due to the sensory properties of the food was reported the most (60.9 %), followed by restrictive eating due to a lack of interest in eating or food (39.1 %) and restrictive intake due to negative consequences of eating (15.2 %). Very little is known about the distribution of ARFID in middle childhood in the general population. One study on eating disorder symptoms in 13-year-old adolescents showed that restrictive eating was present in 2.4 % in girls and 1.8 % in boys, similar to our findings for ARFID. Likewise, in a population-based sample with 5–7-year-old children, 1.4 % of the children were slow/poor eaters based on parent-report [30]. In clinical samples from middle childhood through early adulthood, the prevalence of ARFID was found to be around 14 % [13, 14], which is not surprising given that samples only included participants with an eating disturbance, making them difficult to compare to our sample.

Prevalence data on specific restrictive eating behaviour are scarce. One clinical study found that restrictive eating based on negative consequences such as choking or vomiting (functional dysphagia) is far less common (9 %) than restrictive eating based on the sensory properties of the food (selective eating) (19 %) or on an emotional disturbance (food avoidance emotional disorder) (29 %) [11]. Up to date prevalence studies with non-clinical samples on specific restrictive eating disturbances (except for selective eating) in middle childhood are missing. As different restrictive eating disturbances fall into the diagnosis of ARFID, it is indispensable for the understanding of ARFID, to further investigate its nosology and its prevalence.

Consistent with the DSM-5 [7], ARFID features were equally common in boys and girls. Further, symptoms of ARFID were equally distributed among younger and older children, whereas younger children scored higher on the EDY-Q mean score than older children. This could mean that younger children differ in terms of the specific presentation of avoidant or restrictive eating, in that they might report more emotional food avoidance or a greater avoidance to try new foods compared to older children. In fact, it was shown that several eating difficulties are developmentally normal for infants and preschool children [31, 32]. However, the children under investigation should have already outgrown these difficulties at their age. Further research is required to reassess the possible age differences in the presentation of food avoidance or restriction. Moreover, prospective and longitudinal studies are needed to clarify at which age ARFID develops, and whether or not it presents as a risk factor for later-onset eating disorders. Our finding, that underweight children reported symptoms of ARFID more often than normal- and overweight children, reflects the diagnostic criteria of ARFID, which is required to lead to serious clinical consequences such as weight loss or growth impairment [7, 9, 33]. Interestingly, the EDY-Q mean score was higher in both underweight and overweight children compared to normal weight children, which is understandable, given that overweight children often try to lose weight and are therefore reporting restrictive eating disturbances [34, 35]. Alternatively, this result might be biased through social desirability tendencies according to which the children reported what they assumed to be appropriate. Overall, the EDY-Q shows discriminant validity regarding BMI differences.

Missing data were low, indicating that items were well suited for the ages under investigation. Item difficulty indices were medium to high, reflecting a low probability to score high on items. Further, a significant deviation from normal distribution was found, with a significant left skewed and high kurtosis distribution, all of which indicate that the EDY-Q captures serious and rather rare eating disturbances. Finally, internal consistency of the EDY-Q mean score was acceptable but marginally lower than the frequently used threshold value for scale evaluation of Cronbach’s α ≤ 0.70 [36, 37]. Because the EDY-Q is based on three different presentations of ARFID and is therefore heterogeneous, Cronbach’s α will likely underestimate the reliability of the test [38, 39]. Future research should include a factor analysis of the EDY-Q to measure internal consistency based on the extracted subscales.

Convergent validity could be partly documented through associations with ChEDE-Q items on Restraint over Eating, Fear of Weight Gain and Dissatisfaction with Weight with the EDY-Q exclusionary items for ARFID regarding weight and shape concerns. Divergent validity was found since no or only weak correlations between the ChEDE-Q items described above and the EDY-Q diagnostic items for ARFID and the EDY-Q mean score were found. These initial results concerning convergent and divergent validity of the EDY-Q are promising but further validations, using complete psychometric scales, are needed.

One of the strengths of this study is the large community-based sample size. Both German- and French-speaking children from different cantons of Switzerland were included, speaking for good generalizability. Due to the nature of self-report data, the prevalence rates have to be interpreted with caution. Although school-aged children can make accurate estimations with respect to their health using self-report measures [40], overestimation is likely to occur [21]. Other measures on eating and feeding problems often involve parent- rather than self-report, such as the validated Children’s Eating Behaviour Questionnaire (CEBQ; [41] ). However, valid and reliable self-report questionnaires on eating disturbances in childhood exist as well and are widely used for assessing eating behaviours and attitudes in childhood and adolescence [42, 43], for example, the Children’s Dutch Eating Behaviour Questionnaire (DEBQ-C; [43] ) or the ChEDE-Q [21, 23]. Although a diagnosis of ARFID can only be given with expert interviews and data from other sources, such as parents, self-report measures, such as the EDY-Q, have their merit as an easy and fast way to assess children at risk for eating disturbances. Criteria for a valid and reliable self-report instrument for children’s behaviour and attitudes seem to be simplified language, response alternatives, as well as a restricted length of the questionnaire [43]. The EDY-Q is one of the few questionnaires on eating problems in childhood that was developed for children and was not derived from an adult version. Developed to fit children’s behaviour and understanding, the EDY-Q is very short, making it possible for children to stay focused. To clarify the diagnostic sensitivity of the EDY-Q, reliable interviews for ARFID and associated presentations should be established and used in the future. Another limitation is that information on weight and height was based on self-report. Various studies on the validity of BMI using self-reported weight and height have demonstrated that height was overestimated and weight was underestimated, leading to erroneous assignments of BMI categories [44–46]. If this was true in our study, the underweight category may have been smaller and the overweight category larger, which could have led to reduced group differences.

Overall, this study provides initial information on the distribution of early-onset restrictive eating disturbances in middle childhood from the general population. Features of the new DSM-5 diagnosis ARFID were relatively common by self-report. The EDY-Q presents a promising assessment tool for initial screening on the occurrence of eating disturbances in childhood. If diagnostic sensitivity and psychometric properties can be confirmed, children at risk for eating disturbances could be identified, and targeted prevention, as well as early intervention strategies could be developed for them. ARFID presents as an eating disturbance associated with significant weight loss and growth impairment, as well as social and school attendance difficulties [12, 31, 47]. Therefore, although evidence on long-term consequences is missing, these interventions could result in improved health and decreased health care costs. Further studies on the epidemiology of ARFID and its specific presentations in childhood, as well as its long-term specific outcomes, are needed.
